# Tongue Image–Based Diagnosis of Acute Respiratory Tract Infection Using Machine Learning: Algorithm Development and Validation

**DOI:** 10.2196/74102

**Published:** 2025-08-25

**Authors:** Qianzi Che, Yuanming Leng, Wei Yang, Xihao Cao, Zhongxia Wang, Lizheng Liu, Feibiao Xie, Ruilin Wang

**Affiliations:** 1 Institute of Clinical Basic Medicine of Chinese Medicine, Chinese Academy of Traditional Chinese Medicine, No.16, Nanxiao street, Dongzhimen, Dongcheng District, Beijing, 100700, China; 2 Department of Biostatistics, School of Public Health, Boston University, Boston, MA, United States; 3 Department of Mathematics and Statistics, Boston University, Boston, MA, United States; 4 Department of Traditional Chinese Medicine for Liver Diseases, Fifth Medical Center of the Chinese People's Liberation Army General Hospital, No. 100 West Fourth Ring Middle Road, Fengtai District, Beijing, 1000039, China, 1 13811050593; 5 Institute of Engineering and Applied Technology, Fudan University, Shanghai, China; 6 School of Mathematics and Statistics, Central South University, Hunan, China

**Keywords:** tongue diagnosis, human adenoviruses, COVID-19, image feature extraction, machine learning, traditional Chinese medicine

## Abstract

**Background:**

Human adenoviruses (HAdVs) and COVID-19 are prominent respiratory pathogens with overlapping clinical presentations, including fever, cough, and sore throat, posing significant diagnostic challenges without viral testing. Tongue image diagnosis, a noninvasive method used in traditional Chinese medicine, has shown correlations with specific respiratory infections, but its application remains underexplored in differentiating HAdVs from COVID-19. Advances in artificial intelligence offer opportunities to enhance tongue image analysis for more objective and accurate diagnostics.

**Objective:**

This study aims to develop and validate artificial intelligence–based predictive models using tongue image features to differentiate COVID-19 from adenoviral respiratory infections, thereby improving diagnostic accuracy and integrating traditional diagnostic methods with modern medical technologies.

**Methods:**

A total of 280 tongue images were collected from 58 patients with COVID-19, 84 patients with HAdVs, and 30 healthy controls. Deep learning methods were applied to extract tongue features, including color, coating, fissures, papillae, tooth marks, and granules. Four machine learning classifiers, logistic regression, random forest, gradient boosting model, and extreme gradient boosting, were developed to differentiate COVID-19 and HAdV infections. The key features identified by the machine learning algorithms were further visualized in a 2D space.

**Results:**

Nine tongue features showed significant differences among groups (all *P*<.05), including coating color (red, green, and blue), presence of tooth marks, coating crack ratio, moisture level, texture directionality, roughness, and contrast. The extreme gradient boosting model achieved the highest diagnostic performance with an area under the receiver operating characteristic curve of 0.84 (95% CI 0.78-0.90) and an area under the precision-recall curve above 0.70. Shapley additive explanations analysis indicated tongue color, moisture, and texture as key contributors.

**Conclusions:**

Our findings demonstrate the potential of tongue diagnosis in identifying pathogens responsible for acute respiratory tract infections at the time of admission. This approach holds significant clinical implications, offering the potential to reduce clinician workloads while improving diagnostic accuracy and the overall quality of medical care.

## Introduction

Human adenoviruses (HAdVs) are prominent respiratory pathogens affecting individuals of all ages, leading to acute upper and lower respiratory tract diseases, such as pneumonia and bronchitis [1]. Nearly half (45.7%) of acute respiratory tract infection (ARTI) outbreaks between 2009 and 2020 were attributed to HAdV-7 in China [2]. Since 2020, a temporary decline in HAdVs activity has been noted due to the rapid global spread of COVID-19 and subsequent public health measures [3,4]. As COVID-19 persists in its global circulation, a resurgence of HAdVs and other seasonal respiratory virus infections has occurred [5]. The clinical presentations of ARTI caused by HAdVs and COVID-19 are similar, often presenting as mild symptoms such as fever, rhinorrhea, cough, and sore throat, which poses a significant diagnostic challenge in the absence of viral testing. HAdVs are more prone than COVID-19 to causing acute respiratory infections in children, including pharyngitis, tonsillitis, pharyngoconjunctival fever, bronchitis, and pneumonia [6]. Moreover, recent studies indicate that mixed respiratory viral infections may lead to more severe disease outcomes than single infections [7]. Given the tendency of many patients with ARTI to self-medicate, it is essential to develop methods to differentiate between these common viral infections. Such differentiation could promote timely medical consultation and help reduce the transmission of adenovirus to children.

Tongue image diagnosis is a straightforward, noninvasive, and valuable diagnostic method used in traditional Chinese medicine (TCM), which assesses key features, such as the color, size, and shape of the tongue, along with the color, thickness, and moisture level of the tongue coating [8]. In respiratory infections, TCM theory associates specific tongue coatings with distinct syndromes [9]. Epidemiological studies have also identified distinctive tongue features in patients with acute respiratory infections. Patients with COVID-19 have been reported to present features, such as fissured tongue and strawberry tongue [10-13], whereas tongue signs in HAdV infections remain scarcely documented, limiting comprehensive understanding. Given the convenience and cost-effectiveness of tongue imaging, its application for differentiating COVID-19 from HAdV infections shows promising potential.

Tongue diagnosis traditionally relies heavily on the experience and observational skills of TCM practitioners to interpret tongue features. However, advances in artificial intelligence (AI) technologies have enabled the extraction of tongue image characteristics, such as texture, color, and coating, facilitating more objective and intelligent tongue diagnosis. Recent studies have applied AI-assisted tongue image analysis in various diseases, including diabetes [14], fatty liver [15], non-small cell lung cancer [16], and gastric cancer [17], combining tongue image features with clinical indicators to develop exploratory disease risk prediction models. Similarly, Zhou et al [18] proposed an automatic multiview disease detection system using tongue images, achieving an average classification accuracy exceeding 95% for breast tumors, heart disease, fatty liver, and lung tumors. Given the limited research on AI-assisted tongue diagnosis for respiratory infections, there is a clear need to explore its potential in this domain. Therefore, this study aims to investigate the application of AI-based tongue image analysis to distinguish COVID-19 from adenoviral respiratory infections, with the goal of improving diagnostic accuracy and integrating traditional diagnostic methods with contemporary medical technologies.

## Methods

### Study Sample

Our study was conducted between November 2020 and January 2021 at the Department of Traditional Chinese Medicine Hepatology, which is in the Fifth Medical Center of the Chinese People’s Liberation Army (PLA) General Hospital. The inclusion and exclusion criteria have been listed in Textbox 1

Textbox 1. **Inclusion and exclusion criteria.**

**Inclusion criteria**

- Participants whose age is between 18 and 80 years old.
- Participants willing to participate in tongue image photography signed an informed consent form.

**Exclusion criteria**

- Individuals who were unable to describe their condition clearly due to mental factors or could not cooperate with the collection of tongue diagnosis images.
- Participants with severe acute complications, such as serious electrolyte imbalance and acidosis; patients with other serious internal diseases, such as tumors, immune system disorders, or hematologic diseases; and individuals taking medications, such as steroids, that affect glucose metabolism.
- Pregnant women and nursing mothers were excluded.

**Figure 1.**
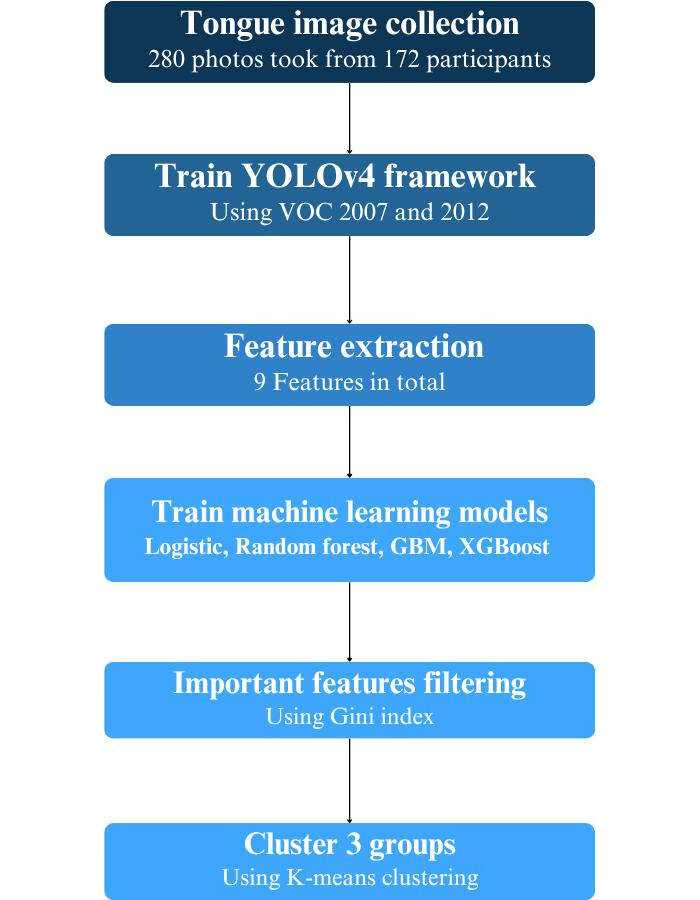
The flow diagram of the participant screening procedure. GBM: gradient boosting machines; XGBoost: extreme gradient boosting.

After applying the inclusion and exclusion criteria, 172 participants were included in the study. Of these, 58 were diagnosed with COVID-19, 84 with adenovirus, and 30 were assigned to the control group. Meanwhile, all the patients had complete clinical data and demonstrated high compliance, while control group participants had no history of chronic or acute diseases in the past 3 months (Figure 1).

### Outcome Definition

A confirmed case of COVID-19 is defined as a suspected case that yields a positive result on a real-time reverse-transcription polymerase chain reaction assay using respiratory specimens [19]. In this study, all patients with adenovirus infection met the diagnostic criteria outlined in the “Adenovirus Infection Diagnosis and Treatment Guidelines" issued in 2013 [20]. According to these guidelines, the criteria include (1) a real-time polymerase chain reaction test on throat swab samples that detects adenovirus-specific nucleic acids; (2) the presence of adenovirus-specific immunoglobulin M antibodies in the serum; and (3) a 4-fold or greater increase in adenovirus-specific immunoglobulin G antibodies in paired serum samples collected during the acute and recovery phases. The selection of diagnostic tests was based on clinical judgment. These tests were conducted on various specimen types, including nasopharyngeal swabs, throat swabs, sputum samples, pleural effusion samples, and bronchoalveolar lavage fluid samples.

### Tongue Image Collection

All tongue images were captured before the laboratory confirmation. Collecting tongue images was conducted either before a meal or 2 hours afterward. Before being photographed, participants were instructed not to eat any food or drink any colored beverages. During the examination, participants were asked to sit with their mouths open and their tongues extended, ensuring the tongue body was relaxed, the surface was flat, and the tip was drooping. A color correction card was held within the camera’s view during image capture to prevent external lighting from affecting the quality of the photograph. If a retake was necessary, the participants were advised to rest for 3‐5 minutes before recollecting the image. The final choice for storage was the best-quality image. A digital diagnostic system was used to collect and analyze all tongue images.

### Tongue Image–Based AI Deep Learning Models

In this study, 4 key factors were primarily selected for detection: the tongue body, the coating, the teeth mark, and the fissures. To develop a target detection algorithm for tongue diagnosis images, the YOLOv4 object detection framework was used [21]. Through extensive experimentation with this algorithm, we used cross-validation to tune the convolutional neural network parameters and found the optimal combination of parameters [22]. We also increased the sample size and improved the model’s generalization using image preprocessing techniques, including random cropping, horizontal flipping, and color distortion [23]. This preprocessing yielded sample images with various tongue characteristics, including color, coating, fissures, prickles, tooth marks, and granules, categorized under data labels 1‐20 (MultimediaAppendices 1  2). Expert TCM practitioners have reviewed all the extracted features to ensure that they possess clinical relevance and significance.

We used VOC 2007 and 2012 to pretrain the initial feature extractor due to its availability and general utility in learning low-level visual features. The training batch size was 32 images, and 2 NVIDIA 1080 8G GPUs were used. Each group was segmented individually for different tongue features, including tongue coating (color, shape, and moisture), fissures (cracks ratio), prickles, tooth marks, and granules (shape and reflectivity).

### Tongue Image Feature Definition

**Figure 2.**
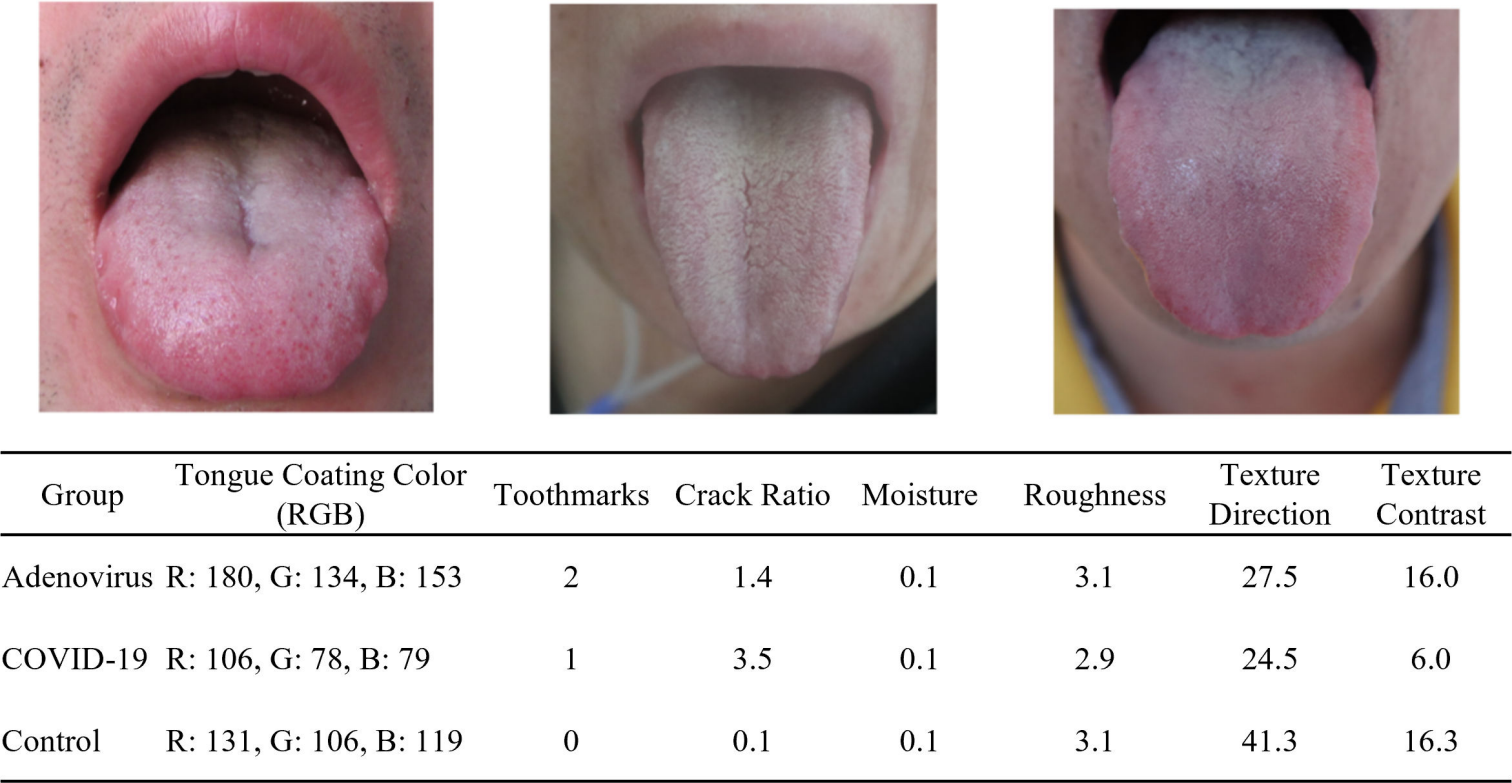
Sample tongue images from the adenovirus (left), COVID-19 (middle), and control group (right) with extracted features.

The color of the tongue coating was calculated by taking the mean red, green, and blue values within a specified area, providing insights into the tongue’s health condition. Tooth marks were identified using the object detection algorithm, with a confidence threshold set at 0.6 to determine the presence and count of tooth marks. The proportion of cracks in the tongue coating was calculated by segmenting and merging areas where multiple fine cracks intersect, thus determining the area occupied by these cracks relative to the total area of the tongue coating. The moisture level of the tongue coating was assessed by identifying reflective areas where brightness exceeds a threshold value of 170, helping to evaluate the hydration status of the tongue. The texture features of the tongue coating were extracted using Tamura texture features, which focus on describing the textural characteristics of the tongue coating in terms of coarseness, contrast, directionality, and roughness [24]. In this study, coarseness refers to texture coarseness and is calculated based on the size of the elements. A larger element size or fewer repetitions of the element indicate a coarser texture. Contrast is primarily derived from the image’s grayscale, while directionality refers to the degree of alignment within the elements (Figure 2).

### Statistical Analysis

We obtained 280 tongue images from these participants, each contributing up to 7 images. Only images taken on the baseline day were retained for this study, and we took the average value of the extracted features in the images if participants had more than one image on the baseline day. There were 21 images with quality issues, including 11 unidentifiable images (all characteristic values were 0) and 10 unclear images (most characteristic values were 0). We applied a mean imputation technique to these images, averaging the image parameters based on outcome category and symptom severity [25]. We conducted comparative analyses across 3 groups to examine variations in tongue features, including color, texture, moisture, and morphology.

The data were divided into 2 subsets, with 70% allocated for training and 30% reserved for internal validation, to address potential overfitting concerns. Advanced machine learning models were used to analyze tongue images and classify them into 3 categories: COVID-19, adenovirus, and control. We used several models in our analysis, including gradient boosting machines (GBM), random forest, logistic regression, and extreme gradient boosting (XGBoost). To fit each model, we applied 5-fold cross-validation on the training set. We evaluated the models’ performance using 2 metrics: the area under the curve (AUC) and the area under the precision-recall curve (AUCPR) [26].

An importance variable rank was computed for each algorithm to identify the variables with the highest predictive power. In addition, we created Shapley additive explanations (SHAP) values that were calculated to indicate the contributing direction of the tongue image features [27]. Finally, we projected the selected variables of tongue images into a 2D space by a t-distributed stochastic neighbor embedding (t-SNE) plot to visually examine each group’s clustering distribution [28].

All statistical analyses were performed using R Studio version 4.2.0 (Posit, PBC) software. An independent samples 2-tailed *t* test was used to analyze the measurement data, such as age and identified tongue image features. The count data were analyzed using the chi-square test. Results with *P*<.05 were considered statistically significant.

### Ethical Considerations

The Ethics Committee of the Fifth Medical Center of the Chinese People’s Liberation Army (PLA) General Hospital (2020074D) gave ethical approval on November 24, 2020. Informed consent was waived by the committee, as the study involved retrospective analysis of anonymized data and did not include any identifiable personal information. All procedures were conducted in accordance with the ethical standards of the institutional and national research committees and with the principles outlined in the Declaration of Helsinki. We ensured that the privacy and confidentiality of all participants were strictly maintained; data were fully anonymized before analysis, and no individual-level identifiers were collected, stored, or reported. No compensation was provided to participants, as the study did not involve direct interaction with human participants.

## Results

### Characteristics of Patients With Each Pathogen

Our research involved 172 participants; 58 had COVID-19, 84 were diagnosed with adenovirus, and 30 were in the control group. Table 1 shows the main descriptive sociodemographic variables (sex and age). The average age in our sample was 23.65 ( SD 9.61) years old (Table 1).

**Table 1.** Study sample and extracted feature characteristics.

Variables		Control (n=30)	COVID-19 (n=58)	Adenovirus (n=84)	*P* value
Gender					
male, n (%)		13 (43)	36 (62)	83 (99)	<.001
Age (years), mean (SD)		27.2 (8.0)	47.6(15.8)	23.7 (9.6)	<.001
Tongue coating, mean (SD)					
Red		119.0 (16.5)	109.8 (22.6)	149.2 (20.4)	<.001
Green		93.3 (13.8)	77.65 (21.2)	110.8 (20.7)	<.001
Blue		97.1 (19.7)	79.2 (22.0)	114.2 (25.4)	<.001
Toothmarks, n (%)					.049
0		20 (67)	20 (35)	33 (39)	
1		6 (20)	17 (29)	24 (29)	
2		4 (13)	21 (36)	27 (32)	
Crack ratio, mean (SD)		0.5 (1.0)	1.4 (2.4)	1.2 (2.0)	.09
Moisture, mean (SD)		0.1 (0.1)	0.1 (0.0)	0.1 (0.1)	<.001
Roughness, mean (SD)		3.2 (0.2)	3.1 (0.2)	3.3 (0.2)	<.001
Texture direction, mean (SD)		40.7 (19.1)	29.9 (17.7)	34.8 (19.1)	.04
Texture contrast, mean (SD)		17.1 (9.4)	12.5 (5.8)	24.6 (9.9)	<.001

### Tongue Image Feature Extraction and Definition

We used transfer learning techniques using weights obtained from training on the VOC 2007 and VOC 2012 datasets, which enabled the model to improve its understanding of general object detection. After 60,000 iterations with a learning rate of 0.0001, the average loss was reduced to 0.2867 (SD 0.0462; Multimedia Appendix 3). Compared to the proposed fast recurrent neural network and faster region-based convolutional neural network, the Single Shot MultiBox Detector architecture demonstrates higher accuracy across 20 labeled datasets (Multimedia Appendix 4). After applying our feature extraction architecture, 9 features of tongue images were identified: tongue coating color values (red, green, and blue), the presence of tooth marks, tongue coating crack ratio, tongue coating moisture level, texture directionality, texture roughness, and texture contrast (Multimedia Appendix 5).

All tongue coating color values (red, green, and blue) differed significantly across groups (*P*<.001) (Table 1). Patients with adenovirus had the highest values, on average, especially in the red component, which may indicate more pronounced inflammation or other specific clinical features related to adenovirus infections. The presence of tooth marks varied across groups, with the highest prevalence of 2 tooth marks in the COVID-19 group, with 21/58 (36%) participants showing this feature (*P*=.049). While the differences may not be statistically significant, both the COVID-19 and adenovirus groups exhibit a notably higher percentage of individuals with 2 tooth marks: 21/58 (36%) participants in the COVID-19 group and 27/84 (32%) participants in the adenovirus group. This is in contrast to the control group, where only 4/30 (13%) participants showed this characteristic (*P*=.09). This may be associated with other symptoms or manifestations of the illness. The moisture levels of tongue coating varied significantly among the different groups, with the adenovirus group exhibiting the lowest average moisture level (*P*<.01).

**Figure 3.**
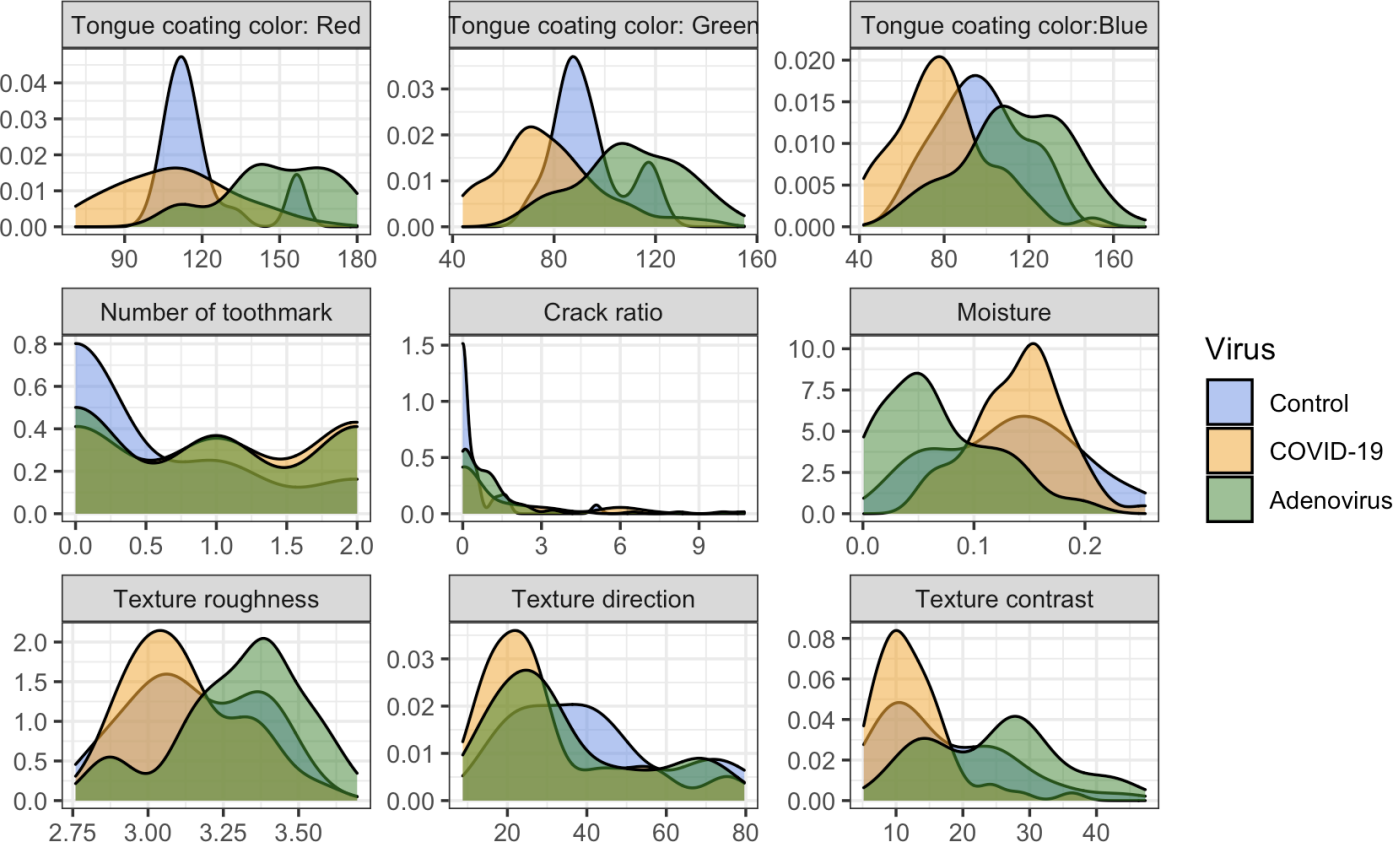
Extracted tongue image feature density plot.

The adenovirus group shows considerably greater levels of tongue coating roughness compared to the COVID-19 and control groups (*P*<.01). In addition, both the adenovirus and COVID-19 groups exhibit significantly greater values in tongue texture direction than the control group, with the adenovirus group showing the highest mean value of 40.7 (SD 19.1). In terms of texture contrast, the COVID-19 group has the lowest average value at 12.5 (SD 5.8), while the adenovirus group reaches the highest average at 24.6 (SD 9.9), in contrast to the control group, which averages 17.1 (SD 9.4). The density plot suggested distinct patterns in tongue features among individuals with COVID-19, adenovirus, and those in the control group, demonstrating the potential of these features in differentiating respiratory viral infections (Figure 3).

### Diagnostic Performance Results

The tree-based and boosting models achieved over 70% AUCPR performance, with AUC exceeding 80% for general performance. The GBM achieved the highest AUC value of 0.888 and AUCPR value of 0.764. XGBoost came in second with an AUC value of 0.872 and an AUCPR value of 0.751, followed closely by random forest with an AUC value of 0.872 and an AUCPR value of 0.747. However, logistic regression showed a relatively worse performance, with an AUC value of 0.812 and an AUCPR value of 0.668 (Multimedia Appendix 6), and the confusion matrix of the test set is presented in Multimedia Appendix 7.

### Explaining the Rationale Behind the Predicted Models

**Figure 4.**
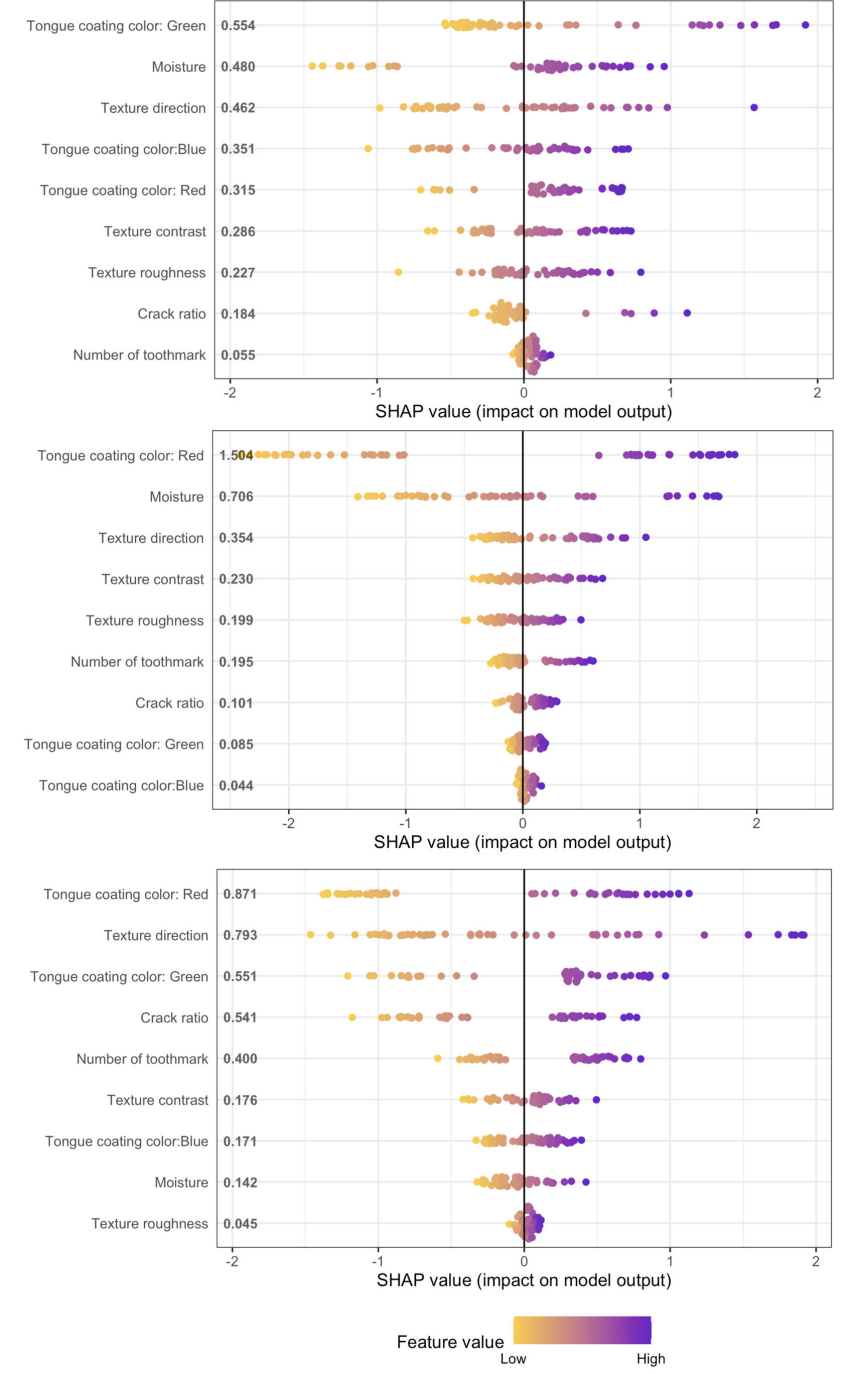
Important features identified for distinguishing between COVID-19 (top), adenovirus (middle), and control (bottom) using SHAP. SHAP: Shapley additive explanations.

**Figure 5.**
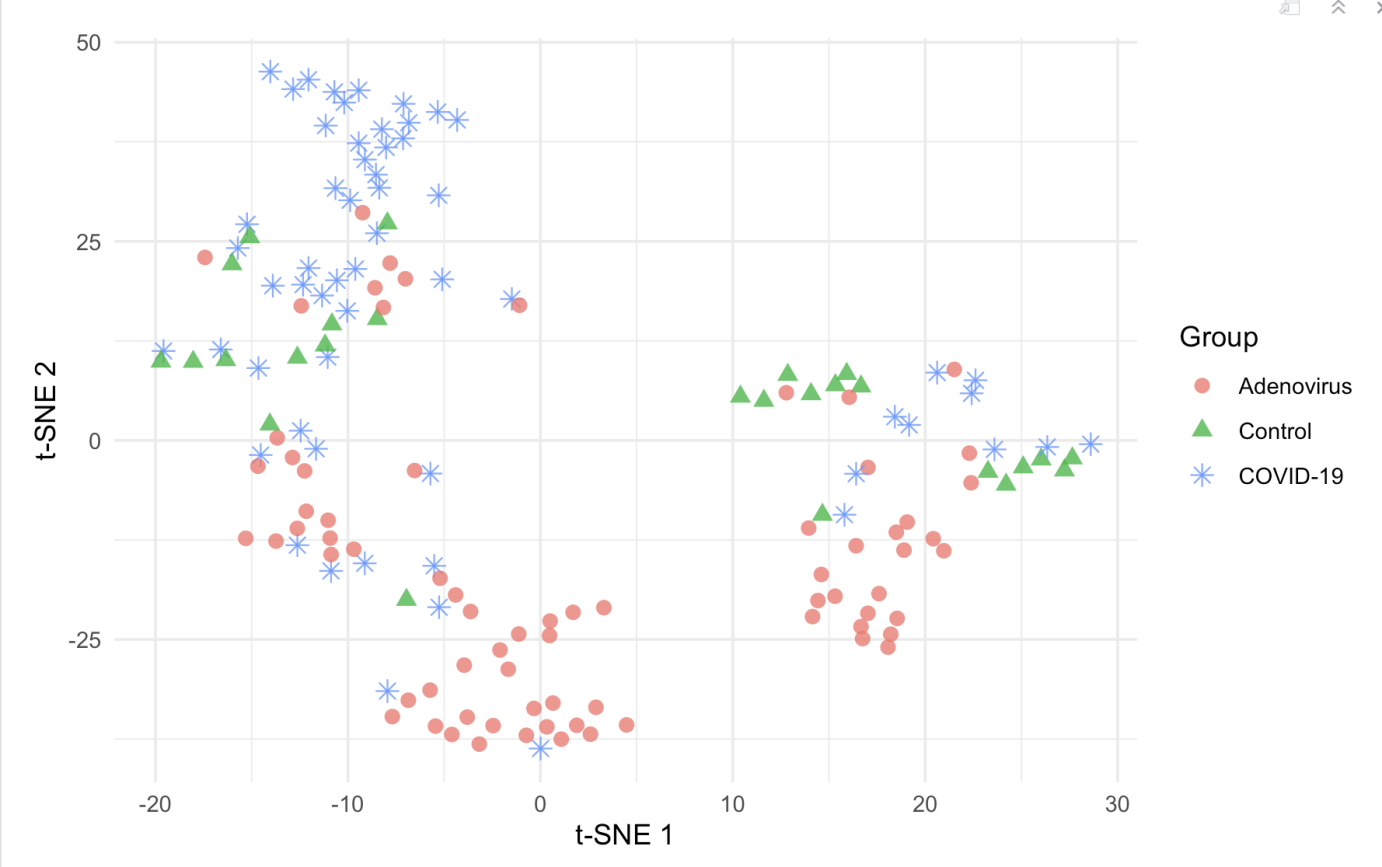
T-distributed stochastic neighbor embedding plot visualizing sample distribution across three groups: adenovirus (circle), control (triangle), and COVID-19 (asterisk), highlighting distinct clustering patterns among the conditions. t-SNE: t-distributed stochastic neighbor embedding.

Based on SHAP values integrating 4 machine learning models, several factors contribute to diagnosing adenovirus and COVID-19, including the color of tongue coating, moisture level, and texture direction. The feature importance plots indicate that the most significant variables are tongue color, moisture level, and texture direction (Multimedia Appendix 8). Specifically, a red tongue coating helps identify adenovirus cases, while a green tongue coating is beneficial for identifying COVID-19 cases (Figure 4). In addition, the t-SNE plot projects these variables into a 2D space and color codes each group. The distribution of patients with COVID-19 and adenovirus differs within the t-SNE space, showing some overlap, while the control group is between the 2 infectious cases (Figure 5).

## Discussion

In this study, we developed and validated an AI-based prediction model to analyze tongue images for the differential diagnosis of respiratory infections caused by COVID-19 and human adenoviruses. Our models demonstrated high diagnostic accuracy, with key tongue image features, such as color, coating, moisture, and texture serving as significant discriminators. These findings highlight the potential of noninvasive tongue image analysis as an effective and cost-efficient diagnostic tool, which may facilitate early and accurate differentiation of respiratory viral infections and support clinical decision-making.

In recent years, the concurrent prevalence of respiratory infectious diseases, such as COVID-19 and HAdVs, has made it challenging to establish a differential diagnosis without pathogenic testing, thereby impacting the efficacy of the treatment [29]. Tongue image diagnosis has emerged as an effective, noninvasive method for auxiliary diagnosis that can be conducted in various settings, catering to the global demands of primary health care systems [30]. In this study, we developed machine learning models that use tongue images to predict acute respiratory tract infection (ARTI) pathogens. The accuracy of these models exceeded 80%, with feature importance plots revealing tongue color, moisture level, and tongue texture direction as pivotal variables. We then projected the tongue image features of the 3 groups of participants into a lower-dimensional space by t-SNE plot. The resulting clusters of COVID-19 and adenovirus were notably distinct, suggesting that the tongue image parameters we extracted contribute significantly to the diagnosis of ARIs. This noninvasive, convenient, and rapid approach has the potential to mitigate unnecessary diagnostic procedures and reduce health care costs.

The constructability of a risk warning model using tongue diagnosis data has been validated in research on metabolic diseases and cancers [16,31]. Our study is pioneering in applying AI deep learning techniques to investigate the diagnostic value of tongue images in acute respiratory tract infection diagnosis and can be integrated into clinical practice to aid decision-making and alleviate physician burden. Previous research by Mai and Krauthammer [32] aimed to predict common respiratory viruses in the United States by combining natural language processing tools with machine learning techniques. However, the model’s prediction performance of HAdVs was moderate, with an area under the receiver operating characteristic curve (AUROC) of 0.53 [32]. Chang et al [33] used an XGBoost model, incorporating demographic, physical examination, laboratory, and vital sign data, to predict HAdVs in hospitalized children with respiratory symptoms, achieving an AUROC of 0.82. Our model exhibited superior performance, achieving an AUROC of 0.87, following the inclusion of various features in its development. In contrast to models established in previous studies, the model developed in this paper benefited from the usage of noninvasive tongue image data, obviating the need for laboratory examination indicators. By using the YOLOv4 object detection framework for image feature extraction and integrating ensemble learning algorithms (ie, GBM and XGBoost), we have demonstrated the clinical utility of tongue images. All 4 models exhibited discriminative validity over tongue images, indicating that tongue images can serve as a reliable tool for ARTI diagnosis and are robust across different AI deep learning model types. Notably, tree-based models, such as GBM, XGBoost, and random forest, outperform logistic regression due to their ability to capture complex nonlinear relationships and interactions among features. Tongue image-derived features often display nonlinear patterns that contradict the linear assumptions of logistic regression. In contrast, tree-based models are nonparametric and resistant to multicollinearity, making them ideal for complex datasets derived from image analysis.

Previous research has validated the use of objective tongue image acquisition equipment, methods, and data analysis techniques [34]. Research on AI in TCM tongue diagnosis has predominantly focused on standardizing tongue diagnosis to minimize human errors [34]. Xu et al [35] developed a multitask joint learning model for segmenting and classifying tongue images, using a deep neural network to optimally extract tongue image features. Meng et al [36] proposed a novel feature extraction framework, termed constrained high dispersal neural networks, to extract unbiased features and minimize human labor in TCM tongue image diagnosis. This study used the YOLOv4 framework, which excelled in extracting high-level image features, thereby enhancing the model’s capacity to identify intricate patterns and subtle variations [21]. The model was initially pretrained on the VOC 2007 and VOC 2012, which are effective for capturing general low-level visual features but lack domain-specific characteristics relevant to medical imagery, such as tongue images. To overcome the challenge of transferring features from natural datasets to specialized domains, domain-specific fine-tuning was performed using collected samples and synthetic data. Although the limited sample size may restrict the model’s ability to capture complex patterns in tongue imagery, the fine-tuned feature extractor achieved approximately 95% classification accuracy across 20 defined groups, demonstrating effective adaptation. This integration, coupled with the fusion of ensemble learning algorithms, harnesses the complementary strengths of clinical and image-derived features, effectively addressing the limitations of each modality in isolation [37]. Consequently, the model gains enhanced discriminative power, leading to more accurate predictions of ARTI.

The analysis of feature importance in machine learning models (GBM, XGBoost, and random forest) revealed that a red tongue coat is most indicative of HAdVs and control group, while the green color is more suggestive of COVID-19 cases. Furthermore, statistical analysis of the tongue images of the 3 groups showed that the COVID-19 group had a higher moisture level and lower contrast rate, suggesting that the patients with COVID-19 are more likely to have a thicker and greasier tongue coating compared to the other 2 groups. Our findings align with the TCM theory. According to TCM, COVID-19 is classified as a “cold-dampness” disease, characterized by a tongue that is pale red or dark and a thick, white greasy coating. HAdVs are considered “warm” diseases in TCM, typically presenting with red and dry tongues. The thickness and dryness of the tongue coating can also indicate the severity of the disease and the extent of fluid damage. Epidemiological studies conducted in China, the United Kingdom, and Ukraine have yielded analogous findings; the predominant tongue colors observed were pale pink and dark red, with the most frequently encountered tongue coating being thin and greasy [38-40]. Therefore, a patient presenting with acute respiratory symptoms and a red tongue devoid of a thick, greasy coating is more likely to have an HAdVs infection. Such observations could guide health care professionals in suggesting additional diagnostic tests and preventive measures, like isolation or improved hygiene, to avert transmission to susceptible household members. Considering its noninvasive nature and rapid assessment capability, AI-assisted tongue image analysis could be particularly valuable as a screening or triage tool in primary care clinics and emergency departments, where timely differentiation of respiratory infections is essential. The advancement of large language models and multimodal AI has facilitated the development of intelligent diagnostic systems that enhance tongue image interpretation and provide real-time decision support. These technologies not only assist clinicians in prioritizing patients for confirmatory testing and early intervention, optimizing health care resource allocation but also enable remote patient monitoring and telemedicine applications.

Hypothetically, the SARS-CoV-2 virus may induce alterations in the expression levels of genes coding for apoptosis and necroptosis of epithelial cells [41,42], resulting in the accumulation of oral epithelial cells and increasing tongue coating thickness. However, these proposed mechanisms remain preliminary and require further experimental validation to clarify their roles in oral manifestations of COVID-19. Supporting the clinical relevance of tongue changes, Wang et al [43] found a correlation between tongue coating thickness in patients with COVID-19 and levels of white blood cells as well as the neutrophil-to-lymphocyte ratio. Conversely, in patients with fever who tested negative for COVID-19, the presence of slimy or greasy tongue fur was associated with the level of C-reactive protein [43]. In addition, studies have indicated that greasy tongue fur is associated with higher blood fibrinogen levels in patients with stroke and with increased activity of glossal epithelial cells and vascular permeability in rodent models [44,45].

It is important to acknowledge several limitations of this study that may impact the generalizability of its findings. Although our predictive models demonstrate the potential of tongue image features for the differential diagnosis of acute respiratory tract infections, their accuracy remains modest, with area under the precision-recall curve (AUPRC) values below 80% across all models (Multimedia Appendix 7). Visualization of the data using t-SNE projected the 3 groups into generally distinct but overlapping clusters in 2D feature space, with considerable overlap, especially between the COVID-19 and adenovirus groups. This overlap is further reflected in our confusion matrix analyses, which consistently show misclassifications between these 2 groups across all classification models. Such overlap between the COVID-19 and adenovirus groups observed in the t-SNE projection may be partly attributable to the similarity of tongue image features shared by these 2 patient populations. In addition, the dimensionality reduction inherent in t-SNE can lead to loss of critical discriminative information, further exacerbating this overlap. Together with the limited sample size used for model development and validation, as well as the exclusion of additional clinical information, such as patient symptoms and other relevant features, these factors collectively contribute to the modest accuracy of our predictive models. Furthermore, the COVID-19 pandemic’s operational constraints necessitated data collection at a single tertiary hospital. Nevertheless, as a national referral center serving Beijing and surrounding provinces, our institution treats patients from geographically diverse regions, which may partially mitigate concerns regarding population representativeness. Future studies should incorporate larger and more diverse cohorts, combined with the integration of comprehensive clinical data, which are warranted to improve the discriminative power and generalizability of the models.

### Conclusion

This study illustrates the utility of AI in helping clinicians identify potential pathogens in ARTI at the time of admission. The interpretability and clinical relevance of our models suggest that they may help reduce unnecessary medical costs and diagnostic procedures while maintaining diagnostic accuracy. Moreover, by alleviating clinicians’ workloads, this approach has the potential to enhance the overall quality of medical care. Looking forward, further research is warranted to externally validate these predictive models in independent cohorts and to expand the sample size to improve generalizability. In addition, incorporating clinical symptoms and other patient-specific features could further refine model performance and support more comprehensive decision-making in respiratory infection diagnosis.

## Supplementary material

10.2196/74102 Multimedia Appendix 1 Tongue image segmentation and feature extraction parameters for 20 labels.

10.2196/74102 Multimedia Appendix 2 Parameters for each layer in the proposed architecture.

10.2196/74102 Multimedia Appendix 3 Iteration loss plot. We iterated 40,000 times with an initial learning rate of 0.001, then reduced it to 0.0001 and continued until 60,000 iterations. Finally, we set the learning rate to 0.00001 and extended the iteration to 70,000.

10.2196/74102 Multimedia Appendix 4 Accuracy comparison among fast recurrent neural network (blue), faster region-based convolutional neural network (orange), and Proposed Single Shot MultiBox Detector (green).

10.2196/74102 Multimedia Appendix 5 Identified tongue image variables.

10.2196/74102 Multimedia Appendix 6 Model assessment matrices for 4 classifiers and 95% CI.

10.2196/74102 Multimedia Appendix 7 Confusion matrix for machine learning model.

10.2196/74102 Multimedia Appendix 8 Mean Shapley additive explanations value over the random forest, gradient boosting machines, extreme gradient boosting. A longer bar indicates the variable is of higher importance in model performance. GMB: Gradient boosting machines.

## Abbreviations

AI: artificial intelligence
ARTI: acute respiratory tract infection
AUC: area under the curve
AUCPR: area under the precision-recall curve
AUROC: area under the receiver operating characteristic curve
GBM: gradient boosting machine
HAdV: human adenovirus
PLA: People’s Liberation Army
RNN: R
SHAP: Shapley additive explanations
t-SNE: t-distributed stochastic neighbor embedding
TCM: traditional Chinese medicine
XGBoost: extreme gradient boosting
