# Supplementary material for: Tongue Image–Based Diagnosis of Acute Respiratory Tract Infection Using Machine Learning: Algorithm Development and Validation
Source: JMIR Med Inform. 2025 Aug 25;13:e74102. doi: 10.2196/74102 (PMC12377515; doi:10.2196/74102)
Supplement: Multimedia Appendix 1 [file medinform-v13-e74102-s001.docx]

**Multimedia Appendix 1.** Tongue image segmentation and feature extraction parameters for 20 labels.

| Labels | 1 | 2 | 3 | 4 | 5 | 6 | 7 | 8 | 9 | 10 | 11 | 12 | 13 | 14 | 15 | 16 | 17 | 18 | 19 | 20 |
| --- | --- | --- | --- | --- | --- | --- | --- | --- | --- | --- | --- | --- | --- | --- | --- | --- | --- | --- | --- | --- |
| Numbers | 80 | 72 | 32 | 30 | 22 | 78 | 80 | 25 | 12 | 94 | 15 | 66 | 11 | 68 | 96 | 48 | 33 | 69 | 75 | 24 |
