# Supplementary figures and images for: Tongue Image–Based Diagnosis of Acute Respiratory Tract Infection Using Machine Learning: Algorithm Development and Validation
Source: JMIR Med Inform. 2025 Aug 25;13:e74102. doi: 10.2196/74102 (PMC12377515; doi:10.2196/74102)

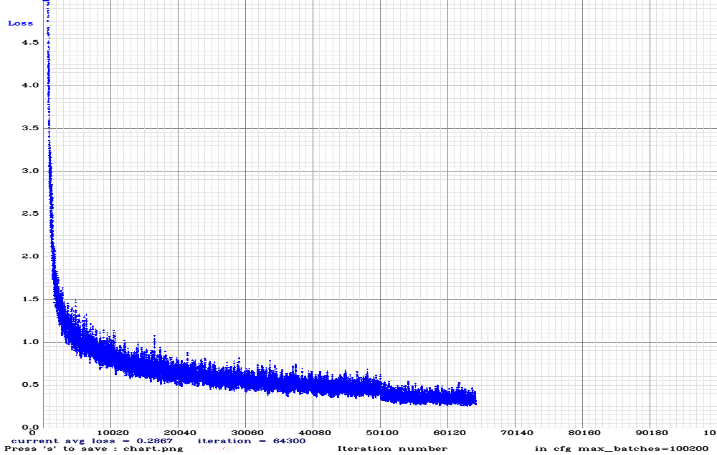

Supplement: Multimedia Appendix 3 [file medinform-v13-e74102-s003.png]

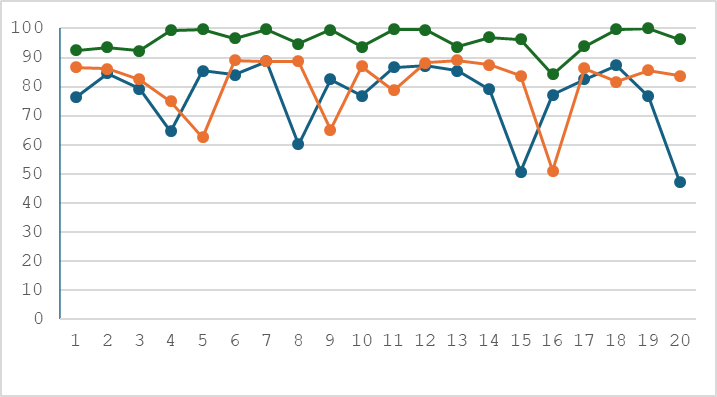

Supplement: Multimedia Appendix 4 [file medinform-v13-e74102-s004.png]

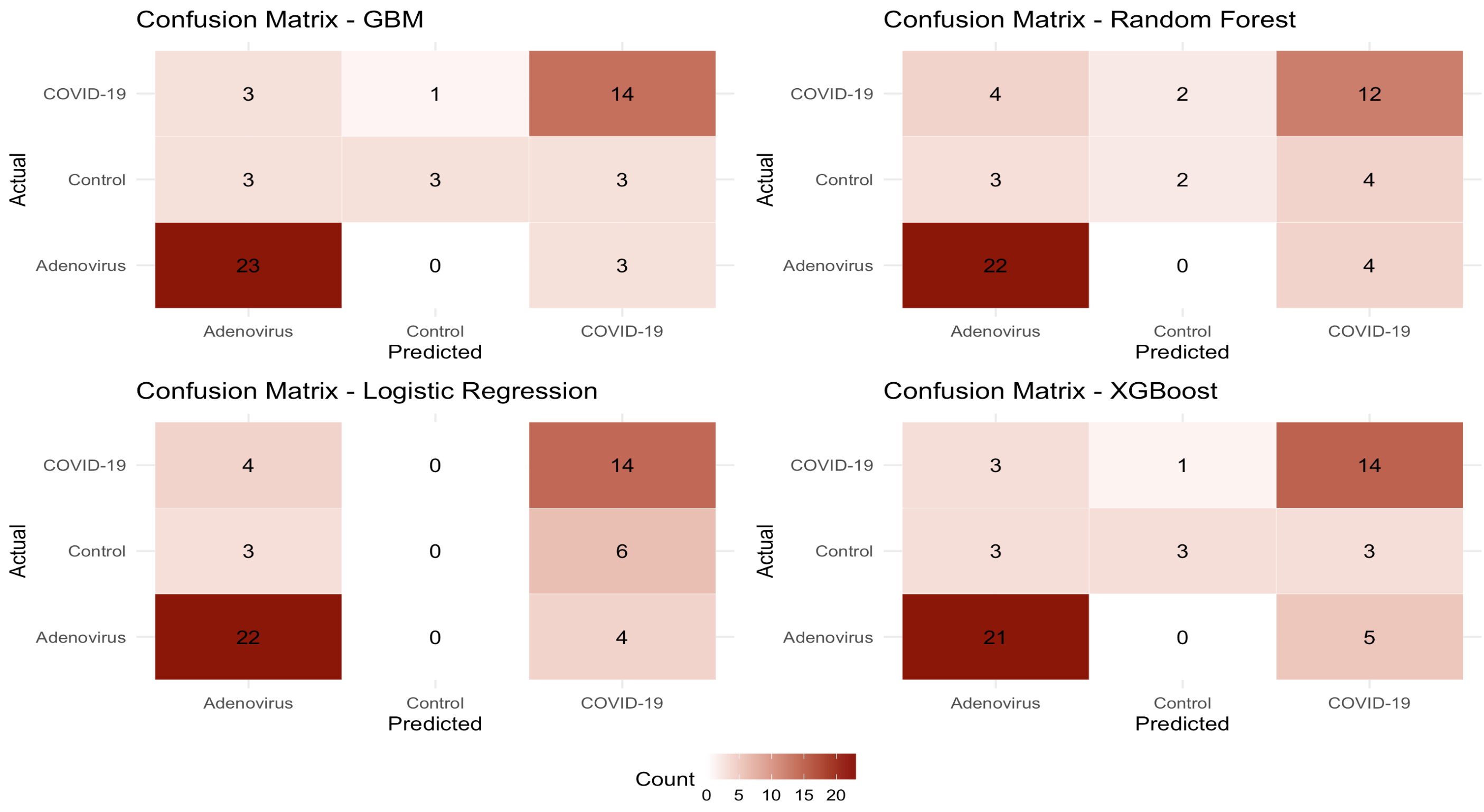

Supplement: Multimedia Appendix 7 [file medinform-v13-e74102-s007.png]

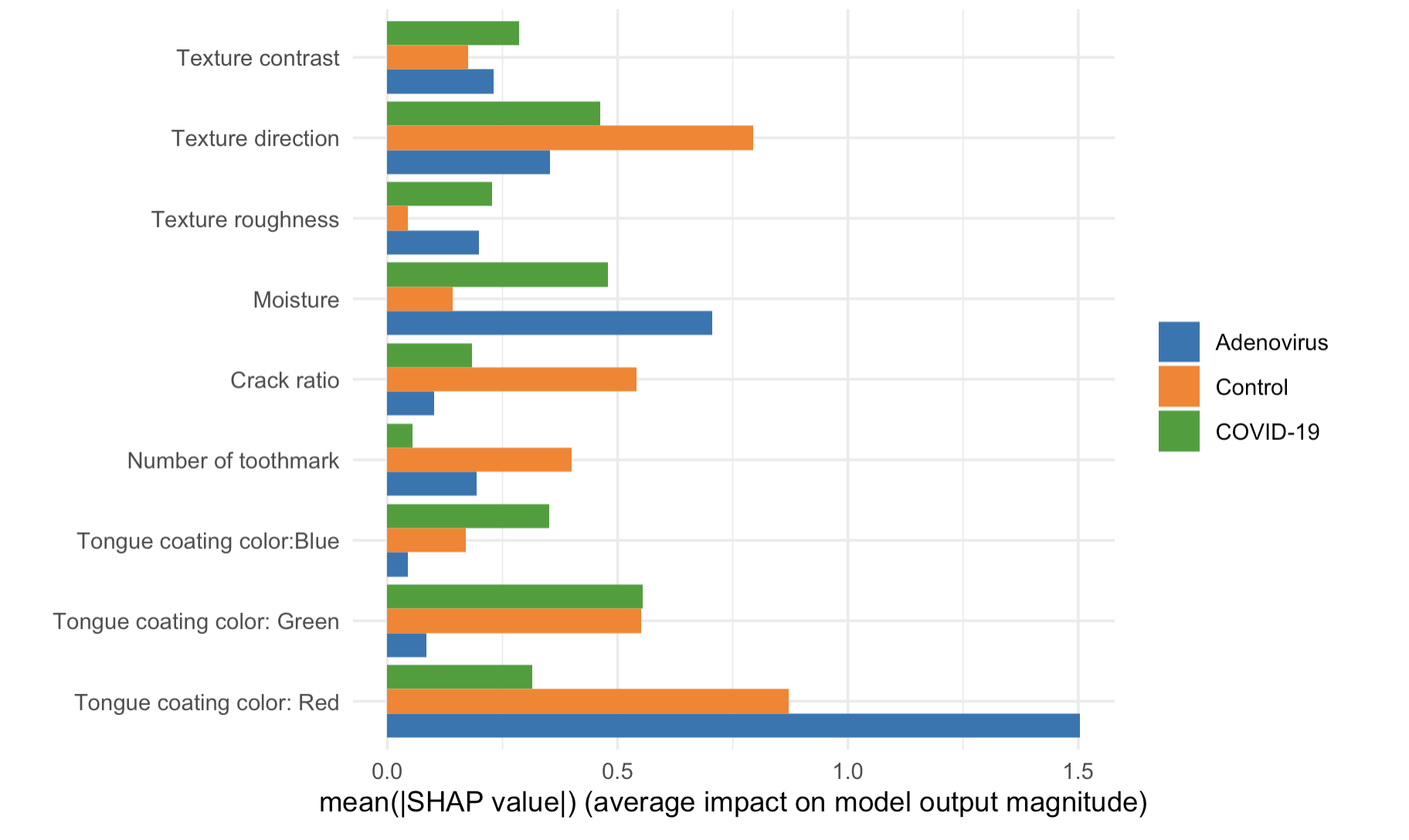

Supplement: Multimedia Appendix 8 [file medinform-v13-e74102-s008.png]
