# Supplementary material for: Tongue Image–Based Diagnosis of Acute Respiratory Tract Infection Using Machine Learning: Algorithm Development and Validation
Source: JMIR Med Inform. 2025 Aug 25;13:e74102. doi: 10.2196/74102 (PMC12377515; doi:10.2196/74102)
Supplement: Multimedia Appendix 5 [file medinform-v13-e74102-s005.docx]

**Multimedia Appendix 5.** Identified tongue image variables

| Feature layers | Conv4_3 | Conv7 | Conv8_2 |
| --- | --- | --- | --- |
| Size of feature layers | 38 × 38 | 19 × 19 | 10 × 10 |
| Anchor sizes | (21, 45) | (45, 99) | (99, 153) |
| Anchor ratios | 2, 0.5 | 2, 0,5, 3, 1/3 | 2, 0,5, 3, 1/3 |
| Prior box | 4 | 6 | 6 |
| Feature layers | Conv9_2 | Conv10_2 | Conv11_2 |
| Size of feature layers | 5 × 5 | 3 × 3 | 1 × 1 |
| Anchor sizes | (153, 207) | (207, 261) | (261, 305) |
| Anchor ratios | 2, 0,5, 3, 1/3 | 2, 0.5 | 2, 0.5 |
| Prior box | 6 | 4 | 4 |
