# Supplementary material for: Tongue Image–Based Diagnosis of Acute Respiratory Tract Infection Using Machine Learning: Algorithm Development and Validation
Source: JMIR Med Inform. 2025 Aug 25;13:e74102. doi: 10.2196/74102 (PMC12377515; doi:10.2196/74102)
Supplement: Multimedia Appendix 6 [file medinform-v13-e74102-s006.docx]

**Multimedia Appendix 6.** Model assessment matrices for 4 classifiers and 95% confidence interval

| Model | AUC | AUC_PR |
| --- | --- | --- |
| Gradient boosting | 0.851 (0.762, 0.939) | 0.782 (0.658, 0.906) |
| Random forest | 0.888 (0.806, 0.969) | 0.713 (0.511, 0.915) |
| Logistic | 0.751 (0.650, 0.851) | 0.414 (0.127, 0.701) |
| XGBoost | 0.907 (0.854, 0.960) | 0.763 (0.556, 0.970) |
